# HairNet: a deep learning model to score leaf hairiness, a key phenotype for cotton fibre yield, value and insect resistance

**DOI:** 10.1186/s13007-021-00820-8

**Published:** 2022-01-19

**Authors:** Vivien Rolland, Moshiur R. Farazi, Warren C. Conaty, Deon Cameron, Shiming Liu, Lars Petersson, Warwick N. Stiller

**Affiliations:** 1 CSIRO Agriculture and Food, Clunies Ross St, Canberra, ACT 2601 Australia; 2 CSIRO Data61, Clunies Ross St, Canberra, ACT 2601 Australia; 3 CSIRO Agriculture and Food, Locked Bag 59, Narrabri, NSW 2390 Australia

**Keywords:** Deep learning, Neural network, Machine learning, Trichome, Hair, Pubescence, Hairiness, Cotton, Leaf, Phenotyping

## Abstract

**Background:**

Leaf hairiness (pubescence) is an important plant phenotype which regulates leaf transpiration, affects sunlight penetration, and provides increased resistance or susceptibility against certain insects. Cotton accounts for 80% of global natural fibre production, and in this crop leaf hairiness also affects fibre yield and value. Currently, this key phenotype is measured visually which is slow, laborious and operator-biased. Here, we propose a simple, high-throughput and low-cost imaging method combined with a deep-learning model, HairNet, to classify leaf images with great accuracy.

**Results:**

A dataset of $\sim$ 13,600 leaf images from 27 genotypes of Cotton was generated. Images were collected from leaves at two different positions in the canopy (leaf 3 & leaf 4), from genotypes grown in two consecutive years and in two growth environments (glasshouse & field). This dataset was used to build a 4-part deep learning model called HairNet. On the whole dataset, HairNet achieved accuracies of 89% per image and 95% per leaf. The impact of leaf selection, year and environment on HairNet accuracy was then investigated using subsets of the whole dataset. It was found that as long as examples of the year and environment tested were present in the training population, HairNet achieved very high accuracy per image (86–96%) and per leaf (90–99%). Leaf selection had no effect on HairNet accuracy, making it a robust model.

**Conclusions:**

HairNet classifies images of cotton leaves according to their hairiness with very high accuracy. The simple imaging methodology presented in this study and the high accuracy on a single image per leaf achieved by HairNet demonstrates that it is implementable at scale. We propose that HairNet replaces the current visual scoring of this trait. The HairNet code and dataset can be used as a baseline to measure this trait in other species or to score other microscopic but important phenotypes.

**Supplementary Information:**

The online version contains supplementary material available at 10.1186/s13007-021-00820-8.

## Background

### A need for accurate phenotyping in research and breeding

Modern agriculture and its associated breeding and research efforts rely on the accurate observation and quantification of key plant traits. Recent years have seen the development of a flurry of image-based phenotyping methods, and a growing number of those integrate recent advances in machine learning and computer vision, and in particular deep learning (for examples, see [1–3]). However, many important traits are still visually scored by humans. Whilst these methods are often simple and well-established, they are prone to human errors, and can be slow and costly. Additionally, if visual scoring is done directly on plants rather than on recorded images, results cannot be revisited when new methodologies emerge.

### Functional importance of cotton leaf hairiness

**Fig. 1:**
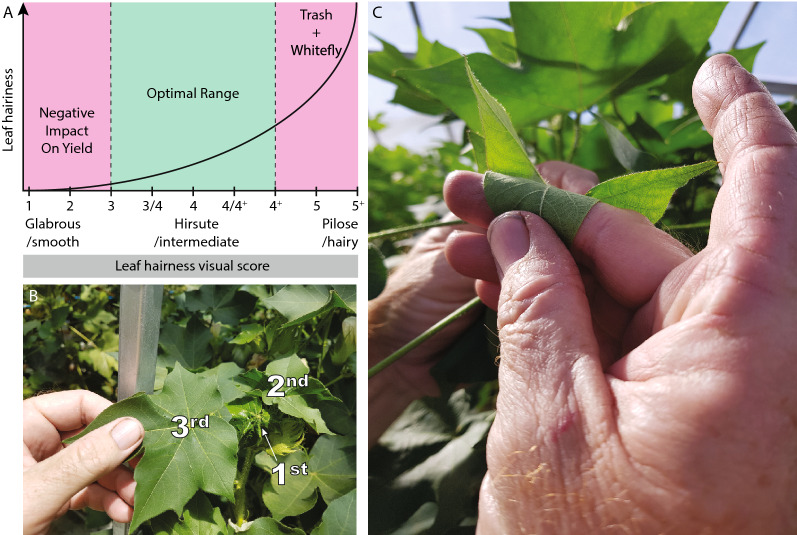
Importance of leaf hairiness and its associated manual scoring method. **A** Leaf hairiness scoring scale highlighting the risks associated with too little or too many leaf trichomes, **B**, **C** manual leaf trichome scoring method typically performed on the 3rd leaf from the top of the plant (**B**) by visually scoring hairiness of the abaxial side of the leaf

Cotton is a food and fibre crop which accounts for 80% of global natural fibre production [4]. The most widely commercially cultivated species of Cotton is *Gossypium hirsutum* L., which is bred across the globe for traits such as improved yield, insect resistance, fibre length and strength, water use efficiency and adaptation to a changing climate. In these breeding efforts, leaf hairiness, also referred to as leaf pubescence, is a key phenotype which is still measured manually [5]. Leaf hairiness is caused by the presence of hair-like cells on the surface of the leaf called trichomes. Trichome structures vary across species and tissues, but globally they regulate leaf transpiration, affect sunlight scattering, and provide a mechanical and chemical barrier against certain insects [6]. In a breeding setting, the importance of measuring cotton leaf hairiness is driven by flow-on effects encountered at both leaf hairiness extremes. On the one hand, the absence (glabrous trait) or low number of leaf trichome are associated with reduced fibre yield [7]. Genotypes with little or no leaf hair are also more sensitive to a range of insect pests such as boll weevil (*Anthonomus grandis*), cotton aphid (*Aphis gossypii*), Asiatic cottonworm (*Spodoptera littoralis*), spotted bollworm (*Earias fabia*), green leafhopper and jassids (*Empoasca* spp.), pink bollworm (*Pectinophora gossypiella*), tobacco budworm (*Helicoverpa virescens*) and several *Lygus* species [8]. On the other hand, genotypes with very hairy leaves (pilose trait) are more susceptible to being colonised by insects such as silverleaf whitefly *Bemisia tabaci* [9]. When cotton is mechanically harvested, high hairiness also promotes gin trash—the accumulation of leaf matter, stalks and dirt in harvested material. Gin trash can downgrade fibre colour and increase the amount of cleaning required prior to ginning, which can negatively affect fibre properties and decrease their economical value [10]. For these combined reasons, an intermediate level of leaf hairiness (hirsute trait) is a highly desirable selection trait for elite cotton varieties (Fig. 1A).

### Current leaf hairiness scoring method

All cotton varieties grown in Australia have been developed by the CSIRO cotton breeding program (Warwick Stiller, pers. comm.). In this breeding program, leaf hairiness is visually scored using an ascending, non-linear 1 to 5+ scale similar to that of Bourland et al, but established over 50 years ago (Fig. 1A) [5, 11]. On this scale, 1 represents the glabrous trait and 5+ is the pilose trait. These two extremes are separated by 7 intermediate scores: 2, 3, 3/4, 4, 4/4+, 4+ and 5. Only genotypes with a leaf hairiness score between 3 and 4+ are selected for subsequent breeding steps.

Scoring a new genotype is currently done by visually inspecting the under (abaxial) side of the 3rd fully unfolded leaf starting from the top of the plant, on six representative plants of that genotype (Fig. 1B, C). Such observations are made in the context of a range of genotypes with well-established scores, representing most levels on the scale and grown under the same environmental conditions as those genotypes which require scoring. A partly subjective decision is made by each observer to integrate the observations made on 6 plants for any given genotype into a single score. In addition to the influence of human interpretation on this method, this technique relies on the reflection of sunlight on trichomes and is therefore only utilised on sunny days. However, until the present study the lack of available alternative has meant that this manual method remains the best approach to score this agronomically important trait.

### Proposed improvement: deep learning-based classification

Recent studies have demonstrated that deep learning approaches can be used to quantify microscopic but important phenotypes such as the density and/or shape of stomata in a range of plant species [12–17]. Quantification of hairiness has been attempted in *Arabidopsis thaliana*, Soybean (*Glycine max*) and Spring Wheat (*Triticum aestivum*) [18–21]. However, these methods require specialised imaging techniques such as 3D X-ray computed tomography or 3D confocal laser scanning microscopy, and/or require time consuming and destructive sample preparation [18–21]. Additionally, none of these techniques leverage the potential of deep learning. To our knowledge, the present study is the first to present a simple imaging method combined with a deep learning approach to quantify leaf hairiness. This method leverages the expertise of crop breeders but offers the robustness and user-independence of machine-learning approaches. More specifically, we used deep Convolutional Neural Networks (CNNs) to transform an input (e.g. a leaf image) into a prediction (e.g. a hairiness score), a task called image classification (for a recent review on deep learning and CNNs, see [22]). This approach relies on having access to data and labels, which in this case are images of cotton leaves and their known hairiness score, respectively. The whole labelled dataset is then split in multiple ways such that the model can be trained and tested, and that a number of aspects of the dataset and model can be investigated.

**Fig. 2:**
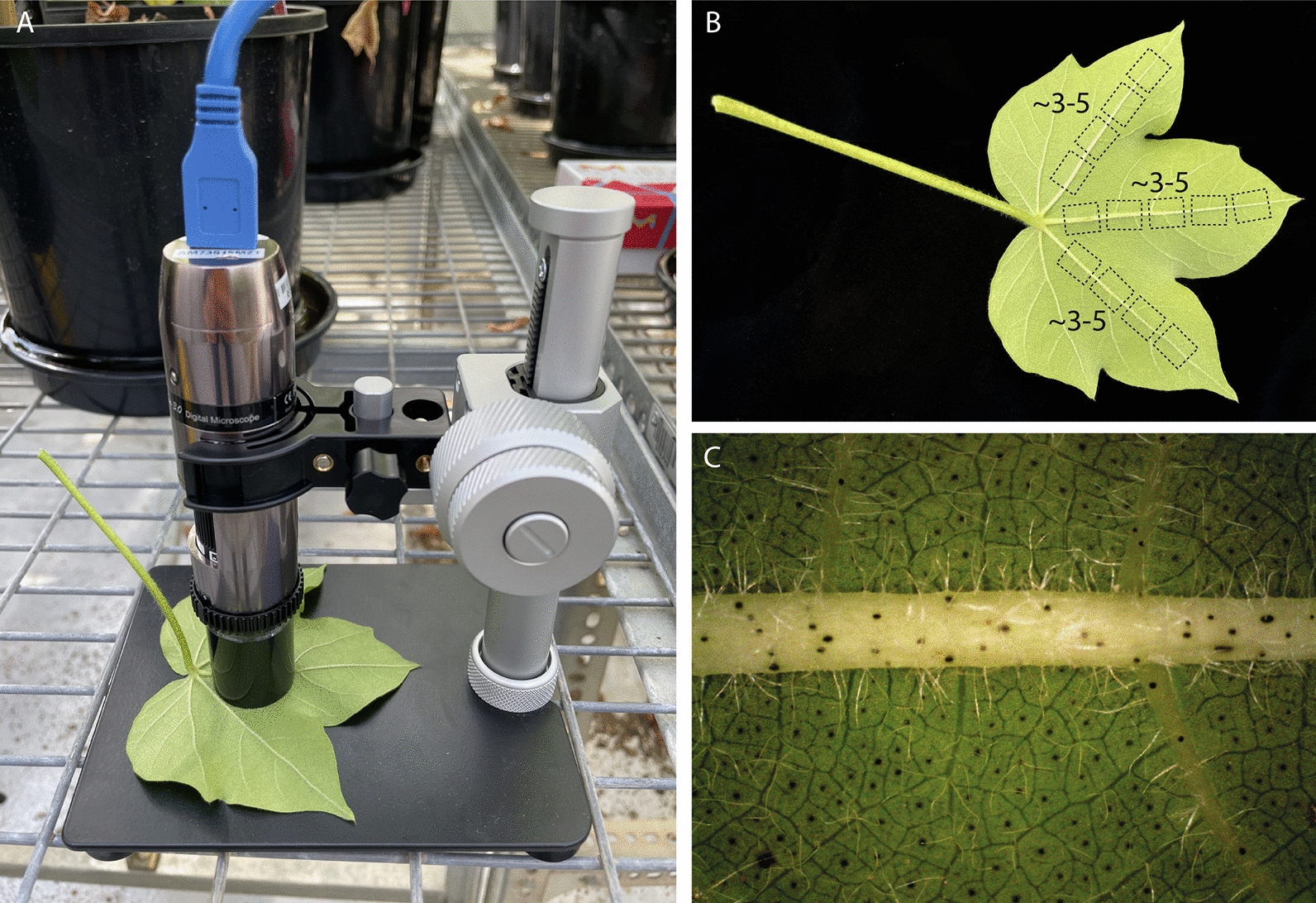
Imaging process and data collection. The abaxial side of the 3rd or 4th leaf from the top was imaged using a hand-held microscope and stage (**A**) and about 3 to 5 images were collected along the 3 main veins of each leaf (**B**) for a total of  9–15 images per leaf (**C**)

**Fig. 3:**
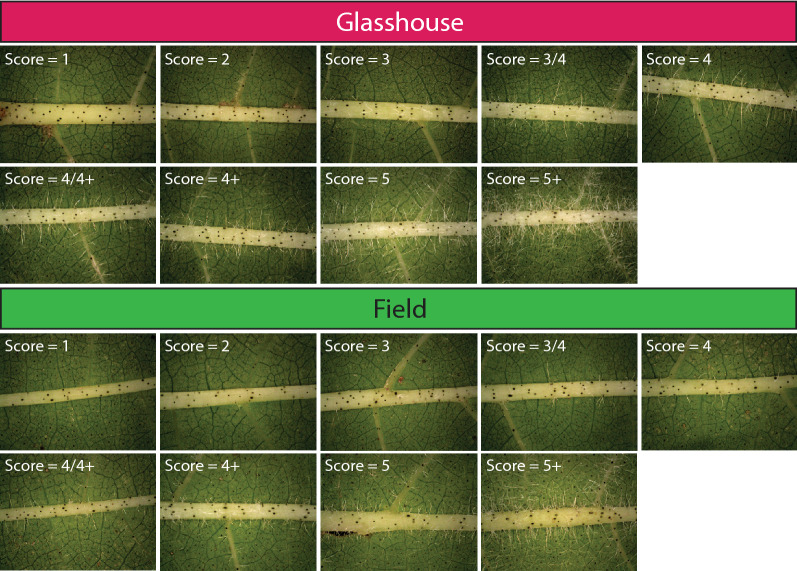
Examples of images taken on Leaf 3 of plants grown in a glasshouse (**A**) or in the field (**B**), for each score class

**Fig. 4:**
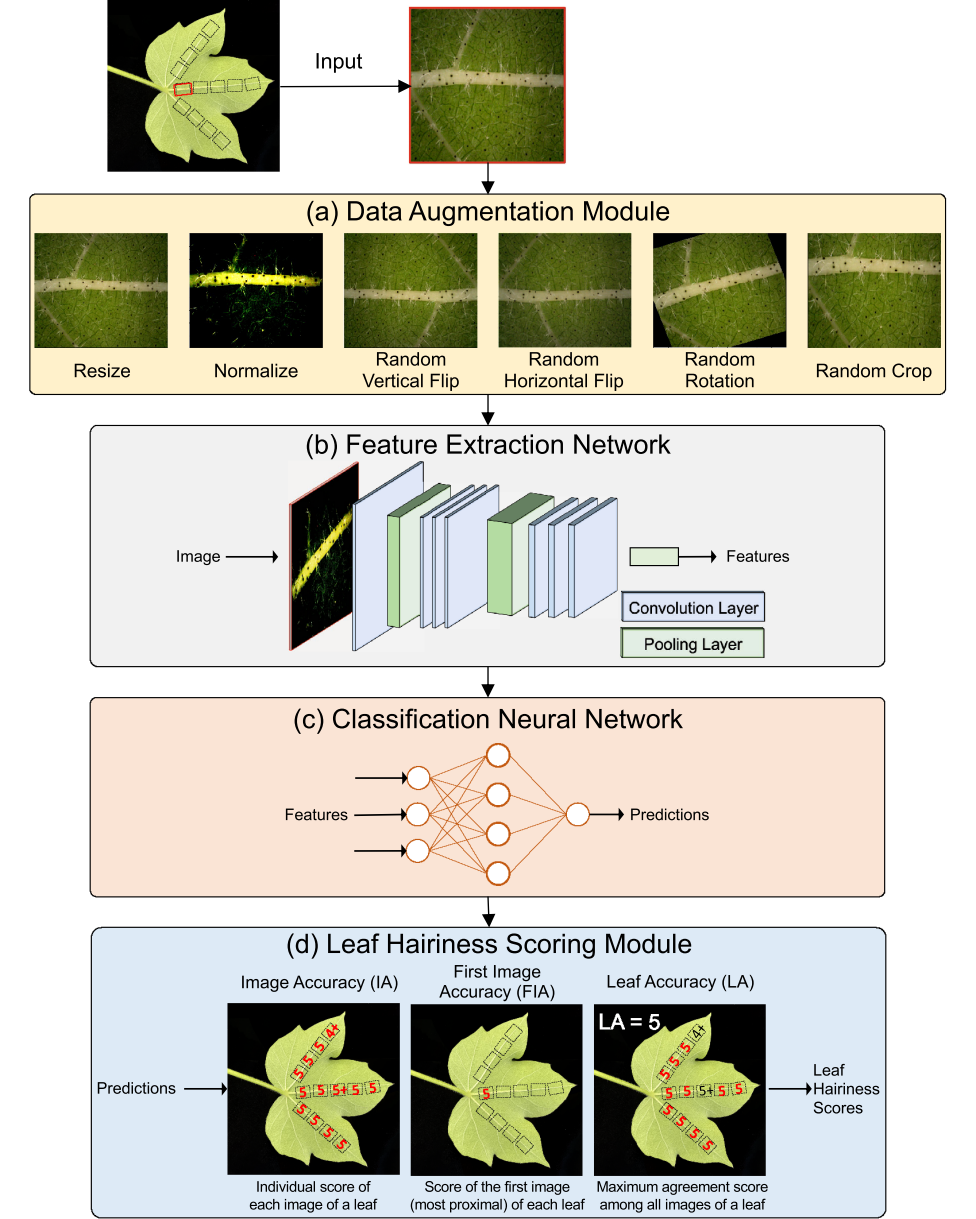
Network architecture of the proposed deep learning model to score cotton leaf hairiness. The proposed model consists of four main parts. First the image is passed through a Data Augmentation module (**a**) that augments the image by applying a variety of image processing techniques. Processed images are then passed to a Feature Extraction Network (**b**) that extracts discriminative visual features from the image representation. Extracted visual features are then passed to a simple Classification Neural Network (**c**) that assigns each input image to a specific score. Raw scores are then processed by the Leaf Hairiness Scoring module (**d**) which generates three accuracy metrics for scoring cotton leaf hairiness

**Fig. 5:**
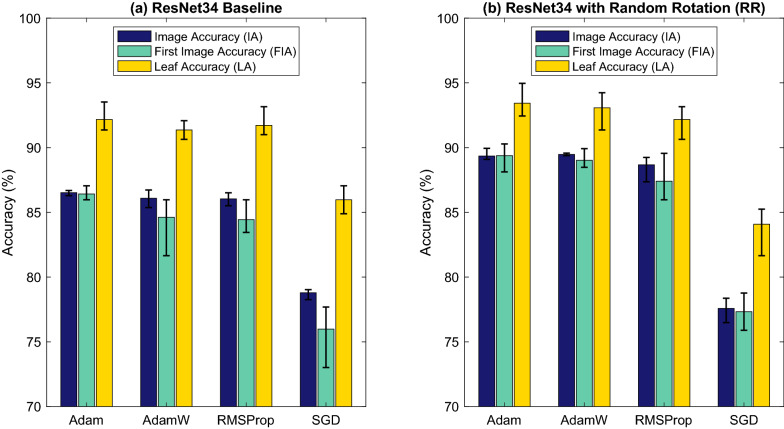
Selecting the best optimizer. Four optimizers were tested with ResNet34 baseline (**a**) and ResNet34 with Random Rotation (RR) data augmentation (**b**), and evaluated on the whole dataset. Error bars represent the maximum and minimum accuracy of four runs of the same experiment

**Fig. 6:**
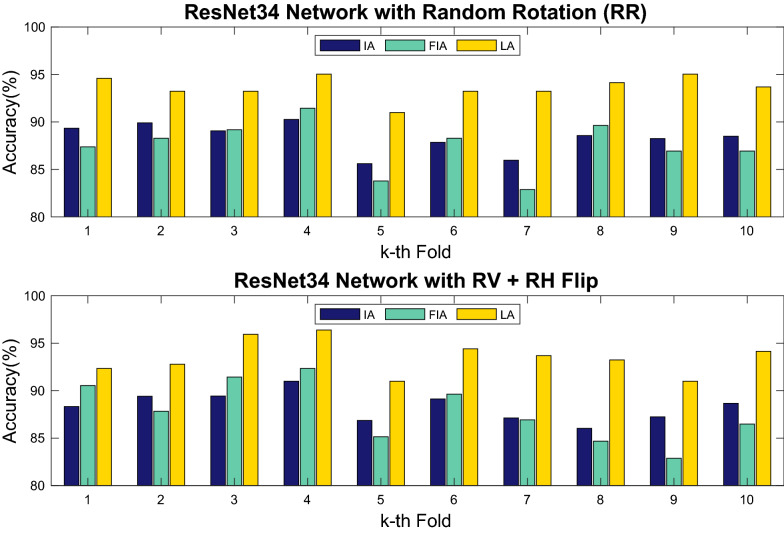
k-fold cross validation. This validation was performed on the training split for the whole dataset with ResNet34 and the two best performing data augmentation techniques (Random Rotation [RR], and Random Vertical and Horizontal Flip [RV + RH Flip]). Here, k was set to 10. IA, FIA and LA refer to Image Accuracy, First Image Accuracy and Leaf Accuracy, respectively

**Fig. 7:**
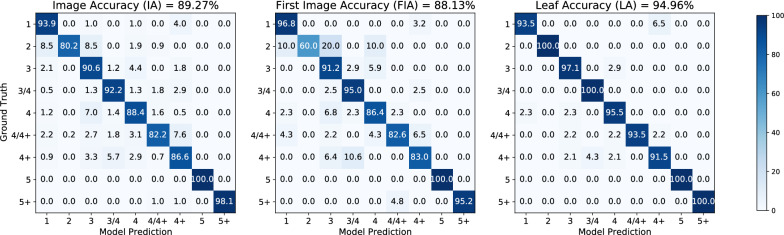
Normalized confusion matrices of the best preforming model on the whole dataset. Image Accuracy (IA), First Image Accuracy (FIA) and Leaf Accuracy (LA) are reported for the best performing model on the whole dataset. The model is able the confidently predict (i.e., strong agreement along the diagonal) the cotton leaf hairiness for all score classes and accuracy measures

**Fig. 8:**
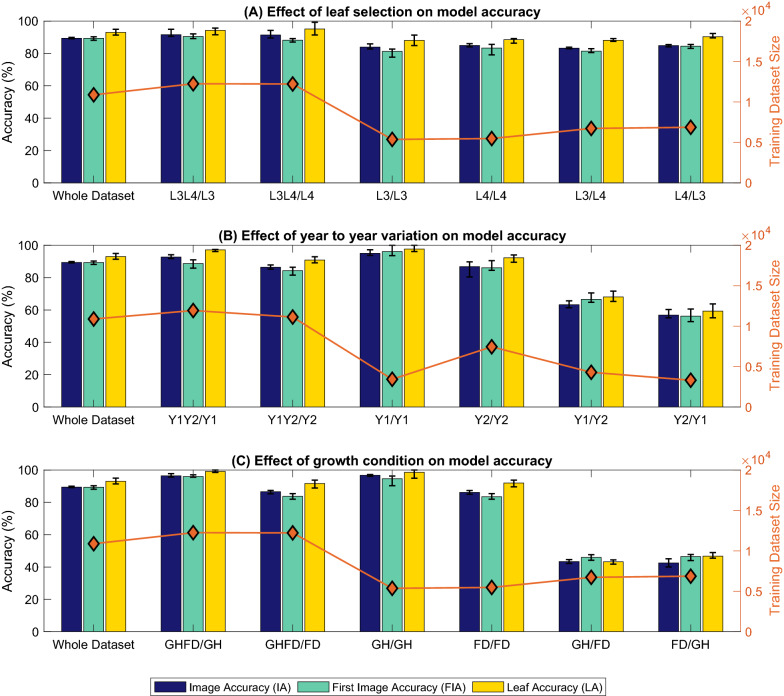
Effect of leaf selection (L3 and L4) (**A**), year to year variation (Y1 and Y2) (**B**) and environment (**C**) on the model accuracy. The best models (ResNet34 feature extractor with Random Rotation (RR) data augmentation and Adam optimizer) performance on the whole dataset is plotted in each row as reference. Each bar in the figure represents the average accuracy and the range clips on each bar represent the maximum and minimum accuracy of five runs of the same experiment with the best model. The training dataset size of each experiment is plotted on the right y axis of each row of bar plots

In this study we hypothesised that an image-based deep-learning model could be used to score leaf hairiness in cotton leaves, accurately and repeatably across genotypes and environments. To this end, we grew a combination of 27 established genotypes in a controlled environment and in the field, and over two successive growth seasons (Table 1); built an image library of 13,597 images (Figs. 2, 3, Table 2); tested different neural network architectures to identify the best approach (Figs. 4, 5, 6, Tables 3 and 4); and finally tested the effect of leaf number (Fig. 8A), growth year (Fig. 8B) and growth condition (Fig. 8C) on the predictions of our best model (Fig. 7). This study is the first to describe an accurate and reliable method to score leaf hairiness, which is a key trait for cotton breeding programs. We propose that our deep learning model replaces the current visual inspection. Additionally, the methodology presented here could be adapted to other crops and plant species. Finally, the image dataset produced for this study is made available for the research community to develop and test novel computer vision approaches.

**Table 1:** Details of leaf hairiness score, site, year and growth condition for each of the 27 genotypes used to create the dataset

Genotype	Score	Year 19–20 (Y1)		Year 20–21 (Y2)		
		FD N	GH N	FD N	GH N	GH C
Pink	1	X	X	X		
Red	1	X	X	X		
Azure	1			X	X	
Charcoal	2			X	X	
Scarlet	3			X	X	
Indigo	3			X	X	
Purple	3	X	X	X		X
White	3/4	X		X		X
Opal	3/4			X	X	
Ebony	3/4			X	X	
Bronze	3/4			X	X	
Orange	4		X			X
Amber	4			X	X	
Emerald	4			X	X	
Copper	4			X	X	
Yellow	4	X	X	X		X
Teal	4/4+			X	X	
Beige	4/4+			X	X	
Green	4/4+	X	X	X		X
Violet	4/4+			X	X	
Crimson	4+			X	X	
Cyan	4+			X	X	
Blue	4+	X	X	X		X
Gray	4+	X	X	X		X
Turquoise	5			X	X	
Brown	5+	X	X	X		X
Black	5+	X	X	X		
Number of genotypes		**10**	**10**	**26**	**16**	**8**

*GH* glasshouse, *FD* field, *N* Narrabri, *C* Canberra

**Table 2:** Number of images and unique scores per growth condition, leaf and year

		Y1	Y2	Whole dataset
				Y1Y2
**Growth condition** (# of images)	GH	2085 (N)	3146 (N) + 1542 (C) = 4688	5231 (N) + 1542 (C) = 6773
	FD	2212 (N)	4612 (N)	6824 (N)
	**Total**	**4297 (N)**	**9300 (N+C)**	**13597 (N+C)**
**Leaf** (# of images/# of leaves)	L3	2062/193	4666/500	6728/693
	L4	2235/193	4364/500	6869/693
	**Total**	**4297/386**	**9300/1000**	**13597/1386**
**Unique scores** (classes)	GH	1, 3, 4, 4/4+, 4+, 5+	1, 2, 3, 3/4, 4, 4/4, 4+, 5, 5+	1, 2, 3, 3/4, 4, 4/4, 4+, 5, 5+
	**Total**	**6**	**9**	**9**
	FD	1, 3, 3/4, 4, 4/4+, 4+, 5+	1, 2, 3, 3/4, 4, 4/4, 4+, 5, 5+	1, 2, 3, 3/4, 4, 4/4, 4+, 5, 5+
	**Total**	**7**	**9**	**9**

*GH* glasshouse, *FD* field, *N * Narrabri, *C* Canberra, *L3* Leaf 3, *L4* Leaf 4

## Methods

### Genotype selection

A total of 27 genetically diverse *Gossypium hirsutum* Cotton genotypes were selected based on their known leaf hairiness to represent the full gamut of observable leaf hairiness variations. Genotype names were anonymised to protect germplasm intellectual property (see details below). Various combinations of these genotypes were grown at two different Australian sites (Narrabri, New South Wales & Canberra, Australian Capital Territory), in the field or controlled glasshouse environment, and over 2 years (2019–2020, referred to as year 1, and 2020–2021, referred to as year 2). For details refer to Table 1.

### Plant growth

#### Field experiments—Narrabri

Plants of 10 and 26 genotypes, respectively, (see Table 1) were established in the summer growing seasons of 2019–2020 and 2020–2021 at the Australian Cotton Research Institute (ACRI, − 30.21, 149.60), 22 km north-west of Narrabri New South Wales, Australia. Seeds of each genotype were planted on Oct. 21 2019 and Nov. 6 2020, at planting density of 10–12 plants m-2 in rows spaced at 1 m. Each genotype was grown in a single 13 m row.

The study region is semi-arid, characterised by mild winters, hot summers and summer-dominant rainfall patterns, with an annual average precipitation of 646 mm [23]. The soil of the site is a uniform grey cracking clay (USDA soil taxonomy: Typic Haplustert; Australian soil taxonomy: Grey Vertosol). Plant available soil water to 1.2 m at the site is between 160 and 180 mm [24]. The soil at ACRI is generally 60 to 65 per cent clay fraction, of low drainage rate [25], pH range of 8.0 to 8.8, and low in organic matter and nitrogen [26].

Nitrogen was applied as anhydrous ammonia approximately 12 weeks before planting at a rate of 200 kg N ha^−1^. Experiments were planted following an 11-month fallow period which was preceded by a winter wheat crop. Management for all field experiments followed current high-input commercial practices: fully irrigated conditions with careful weed and insect control [27]. Plants were furrow irrigated every 10 to 14 d (approximately 1 ML ha^−1^ applied at each irrigation) from December through to March, according to crop requirements. Each experiment was managed according to its individual requirements for irrigation and pest control, with all plots receiving the same management regime. In season 2019–2020, these plants were imaged on the 7th–8th of January 2020 (11 weeks after sowing), whilst in season 2020–2021 they were imaged on the 8th–12th of January 2021 (9 weeks after sowing).

#### Glasshouse experiments—Narrabri

Plants were grown in temperature-controlled glasshouses at the Australian Cotton Research Institute (ACRI). About 15 seeds of each genotype (Table 1) were sown in 8 L plastic pots filled with soil on Sept. 6 2019 and Nov. 2 2020, respectively. The soil was obtained from cotton fields at ACRI (see above). To improve the nutrient status of the potting mix 10 g of MULTIgro® (Incitec Pivot Fertilizers, Melbourne, Australia) basal fertiliser was dissolved into the soil before planting. MULTIgro® contains the nutrients N, P, K, S, and Ca at 13.1, 4.5, 7.2, 15.4, and 2.4 percent, respectively. A 10 mm layer of sand was added to the surface of the pots to reduce surface evaporation and assist in seedling emergence. Once emerged seedlings had reached the three-leaf stage, pots were thinned down to two plants per pot. Plants were grown at 18 ${}^{\circ }$C night and 32 ${}^{\circ }$C during the day, under natural light conditions.

In season 2019–2020, these plants were imaged on the 17th–18th of December (14.5 weeks after sowing), whilst in season 2020–2021 they were imaged on the 5th–6th of January (10 weeks after sowing).

#### Glasshouse experiment—Canberra

Plants were grown in temperature-controlled glasshouses at CSIRO Black Mountain Laboratories, Canberra, Australian Capital Territory, Australia (− 35.27, 149.11). Eight seeds of each genotype (Table 1) were sown in 5 L plastic pots filled with potting mix on Nov. 30 2020. The pots were filled with a 60:40 compost:perlite soil mix. Osmocote® Exact Standard 3-4M (ICL Specialty Fertilizers, Bella Vista, Australia) was sprinkled on the top layer of soil before flowering. Osmocote® Exact Standard 3-4M contains the nutrients N, P, K, Mg, Fe, Mn, B, Cu, Mo and Zn at 16, 3.9, 10, 1.2, 0.45, 0.06, 0.02, 0.05, 0.02 and 0.015 percent, respectively. Two weeks after sowing, pots were thinned down to two plants per pot. Plants were grown at 18 ${}^{\circ }$C night and 28 ${}^{\circ }$C during the day, under natural light conditions. These plants were imaged on the 4th of February 2021 (9.5 weeks after sowing).

### Leaf selection and harvesting

Leaves were numbered in ascending number from the tip of the main stem, with the first fully opened leaf called leaf one. Leaves 3 and 4 from ten individual plants were harvested by cutting their petiole in a proximal position. Harvested leaves were placed in paper bags and imaged within the same day. In the 2019–2020 glasshouse experiment, a few plants died or had a missing leaf, in which case there were genotypes for which leaves 3 and/or 4 were harvested from less than 10 plants.

### Leaf imaging

Single leaves were imaged at a magnification of about 31× with a portable AM73915 Dino-lite Edge 3.0 (AnMo Electronics Corporation, Taiwan) microscope equipped with a RK-04F folding manual stage (AnMo Electronics Corporation, Taiwan) and connected to a digital tablet running DinoCapture 2.0 (AnMo Electronics Corporation, Taiwan) (Fig. 2A). Images were captured on the abaxial side of the leaf, along the 3 central mid-veins. An average of 3 to 5 images were captured in a proximal to distal fashion along each one of the 3 mid-veins, yielding a total of about 9 to 15 images per leaf (Fig. 2B). The exact angle of the mid-vein in each image was not fixed. However, either end of the mid-vein was always cut by the left and right borders of the field of view, and never by the top and bottom ones (Fig. 2C).

### Data de-identification process

As the generated dataset contains images and phenotypical information of commercial importance, the publicly available version of our dataset was de-identified. Australian Privacy Principles (APPs) in the Privacy Act 1988 and the Office of the Australian Information Commissioner (OAIC) recommend that any data de-identification process should ensure the removal of direct and indirect identifiers and placement of safeguards against dataset re-identification. Following these recommendations and the de-identification framework proposed by Data61–CSIRO [28] we followed a two-step de-identification protocol by removing identifiers and preventing re-identification.

#### Removing identifiers

First, images and their associated labels were duplicated into a separate location. Second, unique genotype identifiers present in the duplicate dataset were listed and each genotype was assigned a unique colorname (e.g., green, orange, pink). The assignment of genotype to colorname was documented in a ‘de-identification master file’. Third, unique genotype identifiers in the duplicate dataset were replaced with their associated colornames and all relevant image meta-data (e.g., geo-tag, timestamp) was removed. This final duplicate dataset corresponds to the de-identified version of the original dataset and was used in all the experiments presented in this study.

#### Preventing re-identification

To prevent re-identification, the three different parts required to re-identify the dataset were placed in three separate locations, and access to each part was managed. First, the original dataset was placed in a CSIRO secure cloud storage platform which was only accessible to the people involved in this project. Second, the de-identified dataset was released for open-access through the CSIRO data access portal where the de-identified version of the dataset is made available to the community [29]. Third, the ‘de-identification master file’ was placed in a separate CSIRO cloud storage location and access to it was restricted to the dataset creators and contributors. Without access to all three pieces of the puzzle, one cannot link images to commercially important genotype information, therefore preventing re-identification.

### Data augmentation module

The Data Augmentation module takes raw images as input and performs two or more types of data augmentation (Fig. 4a). All images were resized and normalised as follows:

- *Resize* The feature extraction network requires input images to be of a specific square size. To deal with that, input images ($2560 \times 1920$) were resized to $448 \times 448$ by down sampling.
- *Normalise* The feature extraction network was initialized with pretrained weights learned while training the model on the ImageNet dataset [30], and image pixel values were normalized with the mean and standard deviation calculated from millions of images from this dataset. More details is provided in the section describing the feature extraction network.
  In addition to Resize and Normalise, the following additional data augmentation techniques were tested in this study, individually or in combination:
- *Random Vertical flip (RV Flip)* Input images were vertically flipped randomly with a probability of 0.5 after probabilities ranging from 0 to 1 were tested in increments of 0.1 (Additional file 1: Fig. S1).
- *Random Horizontal flip (RH Flip)* Input images were horizontally flipped randomly with a probability of 0.5 after probabilities ranging from 0 to 1 were tested in increments of 0.1 (Additional file 1: Fig. S1).
- *Random Horizontal and Vertical flip (RV + RH Flip)* Combining RV Flip and RH Flip, input images were horizontal and/or vertically flipped, each with a probability of 0.5.
- *Random Crop (RC)* Input images were first resized to an intermediate resolution of $512 \times 512$, and cropped to $448 \times 448$ at a random location.
- *Random Crop and Vertical Flip (RC + RV Flip)* Images underwent a Random Crop operation before a RV flip operation was performed.
- *Random Crop and Horizontal Flip (RC + RH Flip)* Images underwent a Random Crop operation before a RH flip operation was performed.
- *Random Crop, Vertical and Horizontal Flip (RC + RV + RH Flip)* Images underwent a Random Crop operation before a RV + RH flip operation was performed.
- *Random Rotation (RR)* Input images were rotated from the centre of the image by an angle between + 30 and − 30 °C.

### Feature extraction network

Augmented image representations were fed into a deep convolutional neural network (CNN) called Residual Neural Network (ResNet) [31], to extract discriminative visual features (Fig. 4b). ResNet was chosen as the feature extraction network because its use of skip-connections makes it robust to the vanishing gradient problem [31]. To efficiently perform object recognition, ResNet’s last conversational layer generates a discriminative feature representation of the image based on the training data and labels provided. Such discriminative feature representation are encoded with salient information (e.g., shape, color, texture) of the image required to classify the image into a predefined class. The more discriminative these features are in the high dimensional space, the more accurate the model becomes in predicting which class the input image belongs to. However, learning to generate more discriminative features require access to a very large scale dataset (i.e., millions of densely annotated training images) and an expensive training routine.

To circumvent this we used transfer learning, a technique which allows the backbone CNN network to be trained on a classification task using a very large public dataset, and then transfer this learning to the specific task of interest by training the last few layers of the network only [32]. Here, no layer was frozen but the weights of the ResNet network were initialized with weights learned by the same network when classifying millions of images from the ImageNet dataset [30] into 1000 classes. Learning was then refined during training on cotton leaf images. The use of transfer learning has two main advantages in the context of this study. First, the size of our dataset (13,597 images) is relatively small to train deep networks like ResNet to their full capacity. An alternative would have been to train CNNs more shallow than ResNets (i.e., AlexNet [33], VGG [34]) from scratch on our dataset, but this was deemed sub-optimal. Second, initializing weights with pretrained ImageNet weights helps the model be more robust and generalisable. A model only trained on our dataset would have been more sensitive to any out-of-distribution data not present in the training set.

Five ResNet architectures of increasing depth, namely ResNet18, ResNet34, ResNet50, ResNet101 and ResNet152, were empirically tested for best model performance. With each ResNet, the last fully connected layer of the network was removed, and the output of the last convolutional layer is treated as the extracted visual feature from the image and fed to the classification neural network.

### Classification neural network

The output of the feature extraction layers was passed to a classification network (Fig. 4c). This network consists of a single, fully connected layer which takes the extracted visual features as input and outputs a prediction vector. The weights of the fully connected layer were initialised with the standard Kaiming-Uniform method, which performed within a similar and acceptable range compared to other common methods tested (Additional file 1: Fig. S2). The size of the prediction vector is set to the number of classes in the dataset. Predictions generated from the classification network are fed to the Leaf Hairiness Scoring module.

### Leaf hairiness scoring module

Model accuracy was reported per image, per first image and per leaf (Fig. 4d).

#### Image accuracy (IA)

Image Accuracy was calculated by comparing each predicted image score with the corresponding ground truth score. The higher the IA, the better the model was able to predict cotton hairiness scores. The loss function, which measures how far a prediction is from its ground truth, was minimized with respect to this accuracy and is the primary measure of model performance in our experiments. The other two accuracies were derived from IA.

#### First image accuracy (FIA)

For HairNet to become routine practice, it needs to be easily implementable and it is impractical to capture 15 images per leaf in the field or on a large number of samples. Accuracy on a single image per leaf was therefore measured. To be able to perform meaningful and consistent comparisons between leaves, it was key to focus on images collected at the same position. The most proximal image of each leaf was deemed easiest to consider, especially if multiple users were to image leaves in the future. Consequently, the most proximal image of a given leaf was called First Image for that leaf and its prediction accuracy was called First Image Accuracy (FIA).

#### Leaf accuracy (LA)

During visual human inspection, a leaf is considered as a whole and given a single score. Leaf Accuracy (LA) was calculated by assigning a single score to any given leaf based on the predictions made on images of that leaf present in the test set. For images of a given leaf, the most common score was selected and compared to the ground truth to calculate LA. In the event of two scores being predicted by as many images of a given leaf, the smallest score was selected to calculate LA.

### Dataset splits

#### General approach

With the exception of the k-fold cross validation experiment (see following section), all experiments were performed with a two-way split of the data as follows:

- A candidate image list of $I_n$ images to be separated into train and test split was generated.
- $I_n$ images from the candidate image dataset belonging to a given leaf were grouped to create $L_n$ mutually exclusive groups of leaves.
- $80\%$ of $L_n$ were randomly selected, and images belonging to these leaf groups were set as the training set. As all leaf groups have a similar number of images on average, the size of the training set was therefore approximately $80\%$ of $I_n$.
- Images belonging to the remaining $20\%$ of $L_n$ were set as the test set, with the size of the test also being approximately $20\%$ of $I_n$.

A number of splits were created for this study and are explained below.

#### Whole dataset split

This split was built by placing all 13,597 images of the candidate image list and generating 1386 leaf groups. Here, train and test sets were generated irrespective of leaf number, year and environment. This dataset split is referred to as ‘whole dataset’ and was used to determine the best model architecture.

#### Leaf-based splits

In leaf-based splits, images were placed into train and test sets based on the identity of the leaf they originated from (Leaf 3 or Leaf 4). Below is the list of all leaf-based splits generated in this study, where the names before and after the ‘/’ refer to the train and test conditions, respectively. The first two splits are mixed-leaf splits (training on both leaf identities), the middle two are intra-leaf identity splits (training and testing on the same leaf identity) and the last two are inter-leaf identity splits (training and testing on different leaf identities).

- *L3L4/L3* Here, the candidate image list is all images in the unsplit, whole dataset. Images taken on L3 and L4 were separated into two groups. Images in the L3 group were split according to the process highlighted above, with $20\%$ of L3 images set aside for the test set. The remaining $80\%$ of L3 images and all L4 images were placed in the training set. This split generated a training set made of L3 and L4 images, and a test set consisting of L3 images only.
- *L3L4/L4* This split was similar to L3L4/L3, but here $20\%$ of L4 images were placed in the test set with all other L4 images placed in the training set together with all L3 images.
- *L3/L3* Here, candidate images only consisted of L3 images with $80\%$ being placed in the training set and 20% being placed in the test set.
- *L4/L4* Same as L3/L3 but with L4.
- *L3/L4* Here, all L3 images were placed in the training set and all L4 images were placed in the test set.
- *L4/L3* This split is the reverse of L3/L4, where all L4 images were placed in the training set and all L3 images were placed in the test set.

#### Year-based splits

In year-based splits, images were placed into train and test sets based on the year they were grown in, where year 1 (Y1) and 2 (Y2) refer to seasons 2019–2020 and 2020–2021, respectively. Following the nomenclature of leaf based splits, the first two, middle two and last two splits are mixed-year (training on both years), intra-year (training and testing on the same year), and inter-year (training and testing on different years) splits.

- *Y1Y2/Y1* Here, the test set consists of $20\%$ of Y1 images, with the remaining 80% og Y1 images and all Y2 images placed in the training set.
- *Y1Y2/Y2* In this case, the test set consists of $20\%$ of Y2 images, with the remaining 80% og Y2 images and all Y1 images placed in the training set.
- *Y1/Y1* Here, only Y1 images are used, with 80% placed in the training set and 20% of images placed in the test set. Importantly, Y1 images belong to 7 score classes only (Table 2). So in this case, the model was only trained and evaluated on these classes.
- *Y2/Y2* Similarly to the Y1/Y1 split, only Y2 images were considered for this split. Y2 images belong to 9 score classes so this split was used to train and evaluate a models on these 9 classes (Table 2).
- *Y1/Y2* The candidate image list for this inter-year split was slightly different than for inter-leaf identity splits because the genotypes grown in Y1 and Y2 covered a different number of unique score classes (7 for Y1 and 9 for Y2, see Tables 1 and 2). To circumvent this problem, the candidate image list was limited to images of genotypes and scores present in both years only. Additionally, this split was used to train and evaluate a model on the 7 score classes common to Y1 and Y2 only. From this reduced candidate image list, the Y1/Y2 split was generated by placing all remaining Y1 images into the train set and all remaining Y2 images into the test set.
- *Y2/Y1* Using the same reduced candidate image list as for Y1/Y2, here all remaining Y2 images were placed into the train set and all remaining Y1 images we placed into the test set.

#### Environment-based splits

In environment-based splits, images were placed into train and test sets based on the environment they were grown in (Glasshouse—GH, or Field—FD). Following the same nomenclature as above, the first two, middle two and last two splits are mixed-environment (training on both environments), intra-environment (training and testing on the same environment), and inter-environment (training and testing on different environments) splits.

- *GHFD/GH* Similar to other mixed-split generation processes, $20\%$ of Glasshouse (GH) images were placed in the test set and all remaining images formed the train set.
- *GHFD/FD* Here, $20\%$ of Field (FD) images were placed in the test set and all remaining images formed the train set.
- *GH/GH* Here, $20\%$ of Glasshouse (GH) images were placed in the test set and the remaining 80% of the GH images formed the train set.
- *FD/FD* In this split, $20\%$ of Field (FD) images were placed in the test set and the remaining 80% of the FD images formed the train set.
- *GH/FD* Here, all GH images were used as the training set and all FD images formed the test set.
- *FD/GH* This split is the reverse of GH/FD, where all FD images were used as the training set and all GH images formed the test set.

## Results and discussion

### Building a large and diverse digital dataset

A range of 27 genetically diverse cotton genotypes with leaf hairiness phenotypes ranging from 1 to 5+ were selected in this study (Table 1). These genotypes comprise historical, commercial and unreleased genotypes which vary in their yield, fibre quality, insect/disease resistance, and water use efficiency. Each score class was represented by 1 to 5 unique genotypes, with the most abundant diversity of genotypes in the range that is both the hardest to separate and the most desirable to breeders, namely 3 to 4+. To build a model that works across years and growth condition, these genotypes were grown over two separate seasons (2019–2020—year 1, and 2020–2021—year 2) and in two different environments (Field—FD, and Glasshouse—GH). This is particularly important to reflect the two growth environments used in the breeding pipeline, with some early stages of variety development occurring in the glasshouse and intermediate and later stages being primarily conducted in the field. For each genotype, a total of 10 plants were imaged each year and in each environment (Fig. 2). For each leaf, about 9 to 15 images were collected (Fig. 2B) and to test the importance of selecting the correct leaf, both leaf 3 (L3) and leaf 4 (L4) were imaged. Altogether, this generated a total dataset of 13,597 images split across 1386 leaves, with a full breakdown of the dataset available in Table 2 [29]. Representative images across the scoring scale in the field and the glasshouse are shown in Fig. 3. To protect sensitive germplasm information, the dataset was de-identified and each genotype was renamed after a unique colour.

To train our models, the dataset was either analysed as a whole, or split according to leaf identity (L3/L4), year (Y1/Y2) or environment (GH/FD). In short, each dataset split was prepared by placing 80% of the images of interest into the train set and the remaining 20% of images in the test set. All images of a given leaf were placed in either the test or train set, rather than split across both. Each dataset split follows a ‘train/test’ nomenclature and the size of each split is shown in Table 2, with details about how each split was generated explained in “Methods” section.

### A four part network architecture

To classify leaf images, we used the four part deep learning architecture illustrated in Fig. 4. In this architecture, a raw input image was presented as input to a Data Augmentation module which performed two default operations (normalise and resize), with the benefit of additional operations tested as well (Fig. 4a). Augmented images were then fed to a Feature Extraction Network, where convolution and pooling layers were used to extract discriminative visual features in augmented images (Fig. 4b). Different ResNet network depths were tested and are detailed below. The feature vector generated by the Feature Extraction Network was fed to a Classification Neural Network, which converted its input into class predictions (Fig. 4c). Finally, the Leaf Hairiness Scoring module compared predictions to the ground truth to output a model accuracy (Fig. 4d). Here, three levels of accuracies were generated: for every single image (Image Accuracy—IA), for the most proximal image of each leaf only (First Image Accuracy—FIA) and for each leaf as a whole (Leaf Accuracy—LA).

### Building the best deep learning model

To build the best model, each one of its four parts was optimised starting with the Feature Extraction Network.

#### Selecting a baseline feature extraction network

The performance of five baseline ResNet models with data augmentation limited to *resize* and *normalise* and of growing depths (i.e., ResNet18, ResNet34, ResNet50, ResNet101, ResNet152) was tested on three dataset versions of increasing size and complexity (i.e., Y1/Y1, Y2/Y2 and whole dataset). The three levels of accuracy achieved for each ResNet/dataset combination are reported in Table 3. Overall, the smaller ResNet18 and ResNet34 models achieved the highest accuracy on nearly all combinations, with the exception of FIA on the whole dataset where the deeper ResNet101 performed slightly better (83.09%) than ResNet34 (81.65%). Consequently, ResNet18, ResNet34 and ResNet101 were selected as feature extraction network options in subsequent experiments.

**Table 3:** Selection of visual feature extraction network based on % accuracy scores

Dataset$\rightarrow$	Y1/Y1			Y2/Y2			Whole dataset		
Network $\downarrow$	IA	FIA	LA	IA	FIA	LA	IA	FIA	LA
**ResNet18**	**94.55**	**94.87**	**98.71**	84.00	82.00	**90.50**	83.19	78.41	89.56
**ResNet34**	94.09	94.87	97.43	**84.37**	**83.50**	90.00	**84.85**	81.65	**91.36**
ResNet50	94.43	96.15	98.71	82.43	77.50	88.00	82.60	79.85	89.92
**ResNet101**	93.04	91.02	98.71	82.75	83.00	88.50	82.49	**83.09**	88.12
ResNet152	93.27	91.02	97.43	80.81	74.50	86.00	82.60	79.85	87.76

For each network structure tested, % accuracy scores are reported as Image Accuracy (IA), First Image Accuracy (FIA) and Leaf Accuracy (LA). The highest accuracy in each column and corresponding feature extractor is highlighted in bold. All models here employ the Adam optimizer with learning rate (lr) $=1e^{-4}$

#### Data augmentation technique selection

Data augmentation is the process of performing digital modifications such as rotating, cropping or flipping images on the training set. These techniques can help increase the size of the training dataset and reduce the risks of over-fitting the model [35]. To determine whether data augmentation could help improve the performance of our baseline ResNet18, ResNet34 and ResNet101 models, a range of techniques were tested in the Data Augmentation module (Table 4). Out of the eight modifications considered here, *Random Horizontal and Vertical flip* (RV + RH Flip) and *Random Rotation* (RR) yielded the best accuracies across the three feature extraction networks. Amongst all combinations, ResNet34 + RR had the highest IA (89.27%) and LA (94.96%). ResNet101 + RR was only superior with its FIA (1.8$\uparrow$ compared to ResNet34 + RR). Importantly, ResNet101 has about twice as many training parameters (42.51*M*) as ResNet34 (21.28*M*) and is therefore a slower and more expensive model to run. Taking all the above into account, ResNet34 + RR was considered the overall superior combination and was selected for subsequent experiments.

**Table 4:** Effect of a range of data augmentation techniques on the % accuracy of our three best model architectures, ResNet18, ResNet34 and ResNet101, trained on the whole dataset

Data augmentation $\downarrow$	ResNet18			ResNet34			ResNet101		
	IA	FIA	LA	IA	FIA	LA	IA	FIA	LA
Baseline	83.19	78.41	89.56	84.85	81.65	91.36	82.49	83.09	88.12
RV Flip	87.98	84.17	92.08	88.65	87.56	92.44	85.96	85.25	90.28
RH Flip	87.87	84.53	92.80	88.98	87.12	91.72	84.63	81.65	90.64
**RV + RH Flip**	**88.43**	85.97	**93.88**	89.20	86.69	92.80	87.80	85.97	**93.52**
RC	82.32	80.93	88.48	83.67	81.65	87.05	82.38	83.09	89.20
RC + RV Flip	83.78	83.09	87.76	86.25	84.17	93.16	83.60	82.01	88.12
RC + RH Flip	83.12	81.64	87.76	86.10	84.53	91.72	83.71	82.37	86.69
RC + RV + RH Flip	84.59	85.25	88.84	83.38	81.65	86.33	82.57	82.73	85.97
**RR**	87.80	**87.76**	91.72	**89.27**	**88.13**	**94.96**	**88.72**	**89.92**	92.80

Image Accuracy (IA), First Image Accuracy (FIA) and Leaf Accuracy (LA) are compared to baseline accuracies obtained without data augmentation. The highest accuracy in each column is highlighted in bold. All models here employ the Adam optimizer with learning rate (lr) $=1e^{-4}$

*RV* Random Vertical, *RH* Random Horizontal, *RC* Random Crop, *RR* Random Rotation

#### Optimizer selection

The optimizer is the algorithm which updates weights in the network in response to measurements of the loss function, which calculates the distance between predictions and the ground truth. The experiments presented above were done with the standard Adam optimizer with a default learning rate *lr* of $1e^{-4}$ [36]. No optimization of hyper parameters was carried out on individual experiments enabling more direct comparisons. To test whether another optimizer could improve the performance of our model, the accuracy obtained on ResNet34 and ResNet + RR with Adam was compared to that obtained with another three popular optimizers, namely AdamW, RMSProp and SGD, on the same networks (Fig. 5) [37, 38]. Four experiments were conducted for each combination and the Adam optimizer was found to consistently perform better than AdamW, RMSProp and SGD and was therefore selected as the optimizer for the final model configuration.

#### k-fold cross validation splits

k-fold cross validation experiments can be used to fine-tune model hyper-parameters (e.g., learning rate, batch size) on any split to achieve slightly higher accuracy. However, here we performed this type of experiment with default values to evaluate the sensitivity of our model to different data splits. To this end, the training portion (80%) of the dataset split was used. Using k = 10, the train set was further randomly sampled 10 times to create new 20–80% validation-train set pairs, which each cover 16–64% of the whole dataset. The performance of ResNet34 + RR and ResNet34 + RV + RH Flip on these set pairs is reported in Fig. 6. In both cases, IA, FIA and LA fluctuated within normal range, indicating that there are no detrimental outliers in the data and that our models are robust to handling the natural variation with default parameters.

### Best model performance on the whole dataset

Taking all the above into account, the best model architecture was found to be a Data Augmentation module with *Normalize + Resize + Random Rotation*, a pretrained ResNet34 Feature Extraction Network and a Classification Neural Network with the Adam optimizer (Fig. 4). This deep learning model was named HairNet and trained in 5 independent runs. On the whole dataset the average IA, FIA and LA were 89.40%, 89.35% and 93.02%, respectively (Additional file 1: Table S2 and Fig. 8a). The highest IA, FIA and LA were achieved in two separate training runs and reached 89.94%, 90.28% and 94.96%, respectively (Additional file 1: Table S2). Because of the importance of ascribing a single score to a leaf to reflect the visual assessment currently used in the field, the training run with the highest LA (94.96%) on the whole dataset is thereafter referred to as the best run, or best model. The best HairNet run achieved an IA of 89.27% and a FIA of 88.13%. Normalized confusion matrices of the best model are shown in Fig. 7 and qualitative examples on individual images are presented in Additional file 1: Figs. S3 and S4. The result of the confusion matrices demonstrates that HairNet performs very well across all score classes and that when the model makes an incorrect prediction, it is often not far off the ground truth. For example, the best HairNet model achieved the lowest accuracy on the score class 2, with 80.2% IA (Fig. 7). This score class was particularly hard for the model as it only contains one genotype (‘Charcoal’) which was only grown in year 2020–2021 (Table 1). There was therefore a reduced number of training examples in this class compared to other classes. Despite this challenge, nearly all mispredicted images (17% of the test images) were attributed a predictive score in one of the two neighbouring classes (1 or 3) and only 2.8% of images were attributed another score. This suggests that HairNet is able to learn discriminative features encoding the ordinal relationship between score classes, even in challenging cases.

#### HairNet is robust to Gaussian/white noise

During image acquisition, camera sensors can produce Gaussian noise (white noise) due to a number of factors which include temperature and level of illumination, for example. The effect of Gaussian noise on the performance of the best model was therefore tested by adding to our data varying amounts of noise with random probability, or a fixed amount of noise within a range of probabilities at the data augmentation step. The results of these experiments are presented in Additional file 1: Fig. S5 and demonstrate that HairNet in resistant to Gaussian noise. It is worth noting that the possibility of Gaussian noise being introduced in training or testing images was possibly limited as a result of the precise and robust imaging protocol proposed in this study, which included a constant illumination source within the portable microscope itself.

#### HairNet is versatile and implementable in the field

HairNet achieved a very high IA, which is derived from images taken at any position along the three central mid-veins of leaves. However, for HairNet to be useful in the field or in a breeding program, a high accuracy on a limited number of images per leaf is important. The observation that HairNet achieved similar FIA (88.13%) compared to IA (89.27%) on the whole dataset demonstrates that our model is robust and that imaging could be reduced to only one image per leaf without compromising accuracy. Concurrently, when a human scores plants in the field a single score is given for a whole leaf. The leaf-level accuracy (LA = 94.96%) achieved by the best HairNet run was significantly higher than its associated IA and FIA. This suggests that if imaging is not a limiting step in a given experiment, an even higher scoring accuracy can be achieved. HairNet is therefore a robust model which can easily be adapted to suit the amount of imaging that is practical in a given setting.

### Best model performance on leaf, year and environment-based splits

Whilst HairNet achieved very high accuracies on the whole dataset, the role of leaf selection (Leaf 3 vs. Leaf 4), growth season (year 1 vs. year 2) and environment (Glasshouse vs. Field) on model predictions was next explored. For each variable (leaf, season and environment), the dataset was split in 6 independent ways. Splits where both categories (e.g. L3 and L4) within a variable (e.g. leaf) were represented in the training set are referred to as *mixed* splits (e.g. L3L4/L3, L3L4/L4; where L3L4 represents the training set and L3 or L4, the testing set). Splits where training and testing were done on the same categories are called *intra* splits (e.g. L3/L3, L4/L4). Finally, splits where training and testing were done on different categories are referred to as *inter* splits (e.g. L4/L3, L3/L4). Following this nomenclature the whole dataset split could be referred to as L3L4/L3L4. Each split was used to independently train HairNet five times and the average, minimum and maximum model accuracies on each split, as well as the size of each training set are reported in Fig. 8, Additional file 1: Tables S1, S2 and S3. Furthermore, confusion matrices of the best model performance on each leaf, year and environment-based splits are reported in Additional file 1: Figs. S6, S7 and S8, respectively.

#### Leaf selection does not affect model predictions

As can be seen in Fig. 8a, HairNet performed consistently well on all leaf-based splits and across all score classes as highlighted in Additional file 1: Fig. S6. HairNet even had slightly higher average IA and LA on mixed-leaf splits compared to the whole dataset, whilst FIA remained similar. This is possibly because the training set of mixed-leaf splits was slightly larger to that of the whole dataset due to the presence of all images of the non-test category in the training set. Interestingly, despite their training sets being half the size of the whole dataset split, intra and inter-leaf splits returned very high IA, FIA and LA. This is particularly surprising for inter-leaf splits, where the model was tested on data from a different category to what it was trained on. In this case, the best model still achieved high accuracies of 84% (IA), 83% (FIA) and 89.2% (LA) on L3/L4 and 85.2% (IA), 83.5% (FIA) and 92.4% (LA) on L4/L3 (Additional file 1: Fig. S6). This may be explained by the fact that L3 and L4 have similar appearances and that the model can learn from either, indifferently. Alternatively, because identifying the true L3 and L4 on a plant may sometimes be a little subjective, it is possible that some variation in true leaf identity was introduced by the operator at the time of leaf collection. In any case, these results reinforce the robustness of HairNet, as it was able to perform well on both L3, L4, and mixed leaf populations.

#### Year-to-year variations can be absorbed by mixed training

Similarly to leaf-based split, HairNet returned very high accuracies for mixed-year and intra-year splits (Fig. 8b). With the best model, these high accuracies were observed across all score classes (Additional file 1: Fig. S7). Interestingly, despite a small training set size of about 1/3 compared to the whole dataset split or mixed splits, Y1/Y1 showed the highest model performance with IA = 97%, FIA = 98.7% and LA = 100%. This is possibly due to the fact that in year 1 all examples of a given score class came from the same genotype, which may have reduced the diversity of examples within a class the model learns from. Conversely, despite having much more genotypes in each class, Y2/Y2 yielded high accuracies too, with IA = 89.8%, FIA = 90.5% and LA = 94%. On the other hand, inter-splits were more challenging for the model with Y1/Y2 and Y2/Y1 reaching IAs of 62.9% and 56.9%, respectively. This cannot be solely explained by a reduced training set size due to removal of classes present in one year only, because Y1/Y1 had a similar training set size. Instead, it is more likely due to differences between year 1 and year 2 conditions which may have affected plant characteristics as previously reported [5]. Overall, the key finding of this year-based experiment is that as long as some of the year to be tested was present in the training set, HairNet performed very well. When it comes to implementation, data from a hypothetical year 3 could be tested with known lines and used as is if predictions are good, or slightly adjusted by adding some year 3 data in the training set to refine the model if predictions need to be improved.

#### Environmental variations can be absorbed by mixed training

Similarly to year-based splits, experiments on mixed and intra-environment splits achieved comparable accuracies to those obtained on the whole dataset (Fig. 8c). Accuracies were highest with GHFD/GH (IA = 97.8%, FIA = 95.5% and LA = 100%) and GH/GH (IA = 97.3%, FIA = 94.8% and LA = 100%). This is unlikely due to the size of the training set because for GH/GH it was about half the size it was for the whole set or for GHFD/GH. Instead, it is likely suggesting that leaves grown in the glasshouse were easier to predict than leaves grown in the field, possibly because conditions were more controlled throughout the season and across years in the glasshouse than in the field. Unsurprisingly, HairNet achieved the lowest accuracy on inter-environment splits. On GH/FD and FD/GH, the model achieved IAs of $44.8\%$ and $45.3\%$, respectively. This is likely due to physiological and morphological differences between plants grown in the glasshouse and the field, which remain to be clearly explained but could be influenced by factors such as incidence of insect pressure, wind, and mechanical damage. Interestingly, GHFD/FD performed as well as FD/FD whilst GHFD/GH performed as well as GH/GH (Additional file 1: Fig. S8). From a practical standpoint, this observation suggests that a model trained on a mixed environment (GHFD) is more valuable because it can efficiently predict the score of leaves coming from either environment, which again makes HairNet a robust solution to score leaves in a range of conditions.

## Conclusion

Leaf Hairiness is an important crop trait, which is currently scored manually for a lack of reliable alternative. Here, we have presented a simple imaging set-up to easily capture large amounts of data. We used this dataset to build HairNet, a deep learning model which can automatically score leaf images with a very high accuracy. We showed that HairNet is robust and versatile: we reach 88.13% accuracy when using one image per leaf only and up to 100% accuracy when aggregating scores of multiple images per leaf. Finally, we investigated the variables influencing model performance and found that leaf selection did not influence model outputs, which further improves the robustness of our model. Additionally, variations introduced by different growth seasons and environment did not negatively affect HairNet so long as some data representing the condition to test was also present in the training set. Overall, we demonstrated that this simple imaging set-up combined with HairNet is able to reproduce the current human scoring without being operator-biased whilst offering advantages such as collection of images which can be later revisited. We propose that HairNet replaces the current visual scoring of leaf hairiness. To enable other researchers to fully benefit from our work, we are publicly releasing both the anonymised image dataset [29] and the HairNet code [39] generated in this study (see “Availability of data and materials” section below for URLs). Our image dataset is an important contribution to the science community as a 2020 survey revealed that only 34 field imagery datasets were publicly available, with only 9 of those not relating to fruit detection or weed control [40]. We hope that it will prove useful for the development of a range of data science studies and solutions. The HairNet code can be used as a baseline to measure leaf hairiness in cotton or in other species such as soybean and wheat with simple modifications. Jumping to another species or even another phenotype will require an image dataset tailored to the new task at hand but HairNet may serve as a useful template to build upon.

## Supplementary Information

**Additional file 1: Table S1.** Average [min, max] accuracy of HairNet model on leaf-based splits with training dataset size, as reported in Fig. 8a. **Table S2.** Average [min, max] accuracy of HairNet model on year-based splits with training dataset size, as reported in Fig. 8b. **Table S3.** Average [min, max] accuracy of HairNet model on environment-based splits with training dataset size, as reported in Fig. 8c. **Figure S1.** Effect of varying the probability (p) of Random Vertical (RV) and Random Horizontal (RH) flip data augmentation on model accuracy (Image Accuracy [IA]). **Figure S2.** Effect of different weight initialisation methods in the classification neural network on model accuracy (Image Accuracy [IA]). **Figure S3.** Prediction of HairNet model on random examples from the whole dataset. **Figure S4.** Prediction of HairNet model on random first image (most proximal) examples from the whole dataset. **Figure S5.** Effect of Gaussian noise on model accuracy. **Figure S6.** Normalized confusion matrices of HairNet predictions on leaf-based splits. **Figure S7.** Normalized confusion matrices of HairNet predictions on year-based splits. **Figure S8.** Normalized confusion matrices of HairNet predictions on environment-based splits.

## Data Availability

The HairNet code is available at https://bitbucket.csiro.au/scm/sth/hairnet.git. The HairNet image dataset is available at 10.25919/9vqw-7453.
