# Supplementary material for: HairNet: a deep learning model to score leaf hairiness, a key phenotype for cotton fibre yield, value and insect resistance
Source: Plant Methods. 2022 Jan 19;18:8. doi: 10.1186/s13007-021-00820-8 (PMC8767704; doi:10.1186/s13007-021-00820-8)
Supplement: Supplementary file 1 — Additional file 1: Table S1. Average [min, max] accuracy of HairNet model on leaf-based splits with training dataset size, as reported in Fig. 8a. Table S2. Average [min, max] accuracy of HairNet model on year-based splits with training dataset size, as reported in Fig. 8b. Table S3. Average [min, max] accuracy of HairNet model on environment-based splits with training dataset size, as reported in Fig. 8c. Figure S1. Effect of varying the probability (p) of Random Vertical (RV) and Random Horizontal (RH) flip data augmentation on model accuracy (Image Accuracy [IA]). Figure S2. Effect of different weight initialisation methods in the classification neural network on model accuracy (Image Accuracy [IA]). Figure S3. Prediction of HairNet model on random examples from the whole dataset. Figure S4. Prediction of HairNet model on random first image (most proximal) examples from the whole dataset. Figure S5. Effect of Gaussian noise on model accuracy. Figure S6. Normalized confusion matrices of HairNet predictions on leaf-based splits. Figure S7. Normalized confusion matrices of HairNet predictions on year-based splits. Figure S8. Normalized confusion matrices of HairNet predictions on environment-based splits. [file 13007_2021_820_MOESM1_ESM.pdf]

Accuracy	Leaf-based dataset Split						
	WD	L4L3/L3	L4L3/L4	L3/L3	L4/L4	L3/L4	L4/L3
IA	**89.40**<br>[89.09,<br>89.94],	**91.57**<br>[90.61,<br>94.96],	**91.39**<br>[89.58,<br>94.24],	**83.91**<br>[82.75,<br>85.92],	**84.94**<br>[84.04,<br>86.14],	**83.27**<br>[82.87,<br>83.96],	**84.85**<br>[84.06,<br>85.47],
FIA	**89.35**<br>[88.13,<br>90.28]	**90.49**<br>[89.20,<br>92.08]	**88.19**<br>[87.05,<br>89.20]	**81.29**<br>[77.69,<br>82.73]	**83.30**<br>[79.13,<br>85.61]	**81.40**<br>[80.51,<br>82.97]	**84.50**<br>[83.11,<br>85.56]
LA	**93.02**<br>[91.36,<br>94.96]	**94.28**<br>[91.56,<br>95.68]	**95.11**<br>[91.42,<br>99.28]	**88.05**<br>[84.89,<br>91.36]	**88.48**<br>[86.33,<br>89.21]	**88.16**<br>[87.44,<br>89.18]	**90.38**<br>[89.61,<br>92.35]
Trainset Size	10883	12251	12223	5371	5478	6728	6869

Additional file: Supplementary Table 1
Average [min,max] accuracy of HairNet model on leaf-based splits with training dataset size, as reported in Figure 8a.

Accuracy	Year-based dataset Split						
	WD	Y1Y2/Y1	Y1Y2/Y2	Y1/Y1	Y2/Y2	Y1/Y2	Y2/Y1
IA	**89.40**<br>[89.09,<br>89.94]	**92.73**<br>[91.79,<br>94.17]	**86.43**<br>[85.25,<br>87.88]	**95.04**<br>[93.74,<br>97.21]	**86.82**<br>[80.43,<br>89.78]	**63.28**<br>[61.41,<br>65.64]	**56.82**<br>[55.17,<br>60.27]
FIA	**89.35**<br>[88.13,<br>90.28]	**88.71**<br>[85.89,<br>91.02]	**84.34**<br>[81.52,<br>86.41]	**96.15**<br>[93.58,<br>100.00]	**86.10**<br>[84.50,<br>90.50]	**66.61**<br>[64.72,<br>70.55]	**56.21**<br>[52.84,<br>60.62]
LA	**93.02**<br>[91.36,<br>94.96]	**97.17**<br>[96.15,<br>97.44]	**90.87**<br>[89.13,<br>92.93]	**97.69**<br>[96.15,<br>100.00]	**92.30**<br>[89.50,<br>94.00]	**68.05**<br>[65.27,<br>71.66]	**59.32**<br>[55.18,<br>63.73]
Trainset Size	10883	11935	11129	3434	7450	4297	3315

Additional file: Supplementary Table 2
Average [min,max] accuracy of HairNet model on year-based splits with training dataset size, as reported in Figure 8b.

Accuracy	Environment-based dataset Split						
	WD	GHFD/GH	GHFD/FD	GH/GH	FD/FD	GH/FD	FD/GH
IA	**89.40**<br>[89.09,<br>89.94]	**96.37**<br>[95.67,<br>97.78]	**86.59**<br>[85.27,<br>87.64]	**96.71**<br>[96.09,<br>97.29]	**86.12**<br>[84.99,<br>87.37]	**43.38**<br>[42.20,<br>44.82]	**42.52**<br>[40.02,<br>45.25]
FIA	**89.35**<br>[88.13,<br>90.28]	**95.97**<br>[95.52,<br>97.01]	**83.74**<br>[81.94,<br>85.41]	**94.62**<br>[90.29,<br>96.26]	**83.47**<br>[81.94,<br>85.41]	**45.99**<br>[44.16,<br>47.64]	**46.45**<br>[43.99,<br>47.74]
LA	**93.02**<br>[91.36,<br>94.96]	**99.10**<br>[98.50,<br>100.00]	**91.66**<br>[88.88,<br>93.75]	**98.53**<br>[94.91,<br>100.00]	**91.94**<br>[89.58,<br>93.75]	**43.24**<br>[41.80,<br>44.44]	**46.69**<br>[45.49,<br>48.95]
Trainset Size	10883	12242	12232	5416	5438	6773	6824

Additional file: Supplementary Table 3
Average [min,max] accuracy of HairNet model on environment-based splits with training dataset size, as reported in Figure 8c.

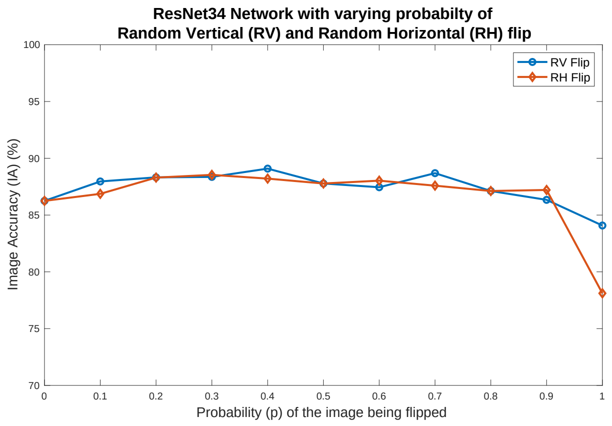

Additional file: Supplementary Figure 1
Effect of varying the probability (p) of Random Vertical (RV) and Random Horizontal (RH) flip data augmentation on model accuracy (Image Accuracy [IA]).
Apart for extreme cases ($p = 0$ or $p = 1$), both RV and RH flip data augmentation returned a similar IA when tested on the whole dataset. At $p = 1$, IA dropped because this data augmentation was applied to all images in the training set which became out of distribution with the testing set. A value of $p = 0.5$ (random) was used in all subsequent experiments.

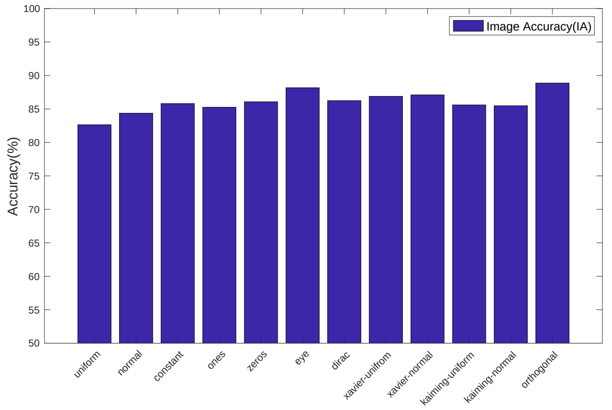

Additional file: Supplementary Figure 2
Effect of different weight initialisation methods in the classification neural network on model accuracy (Image Accuracy [IA]).
As the feature extraction network was initialised with pretrained weights from ImageNet, only the classification layer in the HairNet model was initialised with different techniques to evaluate its effect on the model accuracy. All initialisation methods for the classification layer were found to have little effect on model accuracy. PyTorch's default neural network instillation 'Kaiming-uniform' (named after Kaiming He, author of ResNet model[31]) was used in all experiments.

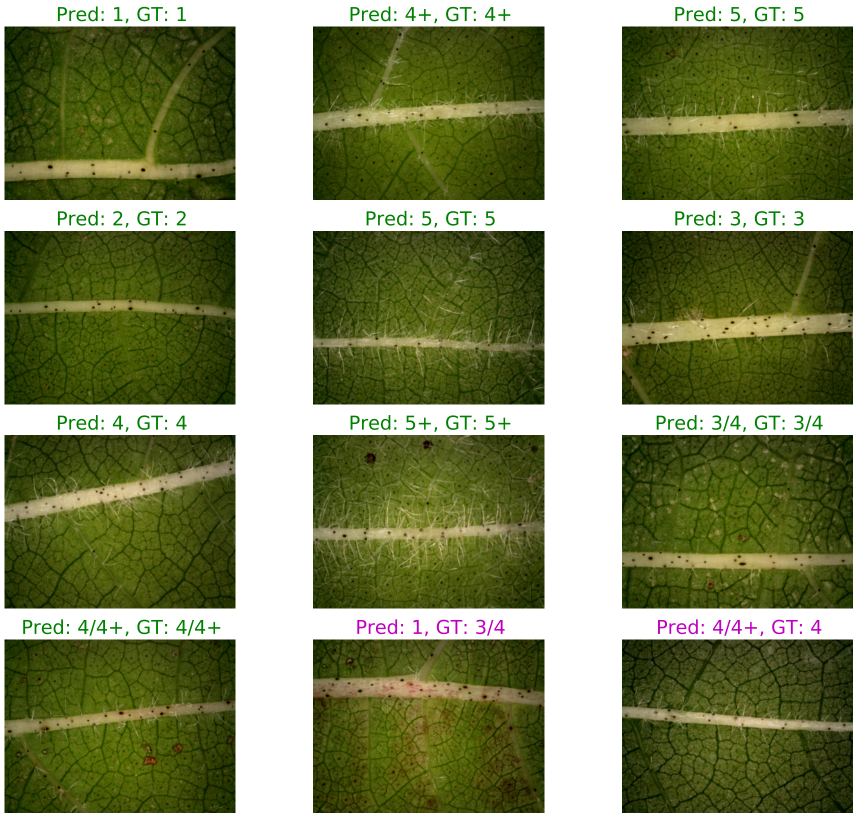

Additional file: Supplementary Figure 3
Prediction of HairNet model on random examples from the whole dataset.
Qualitative results comparing the prediction (Pred.) of HairNet model and corresponding ground truth (GT) on the whole dataset are reported. Correct predictions are highlighted in green, and wrong predictions in magenta. The correct and incorrect examples reported here are chosen randomly from the test set irrespective of growth environment, leaf number, year or image position on the leaf.

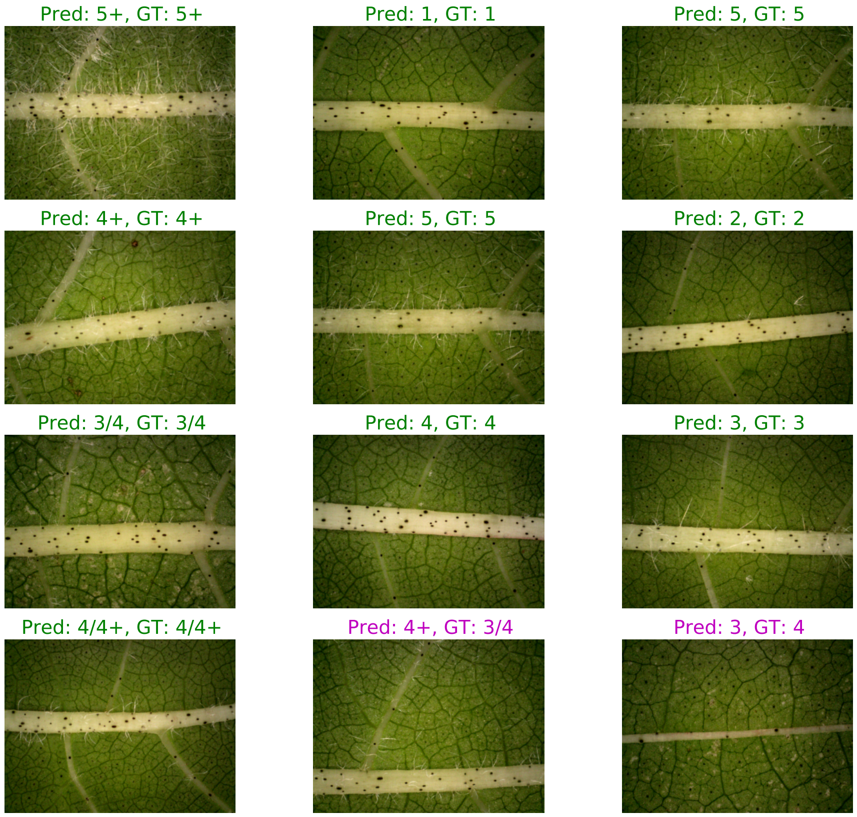

Additional file: Supplementary Figure 4
Prediction of HairNet model on random first image (most proximal) examples from the whole dataset.
Qualitative results comparing the prediction (Pred.) of HairNet model and corresponding ground truth (GT) on only the first images (most proximal) of a leaf from the whole dataset are reported. Correct predictions are highlighted in green, and wrong predictions in magenta. The correct and incorrect examples reported here are chosen randomly from the test set irrespective of growth environment, leaf number, year or image position on the leaf.

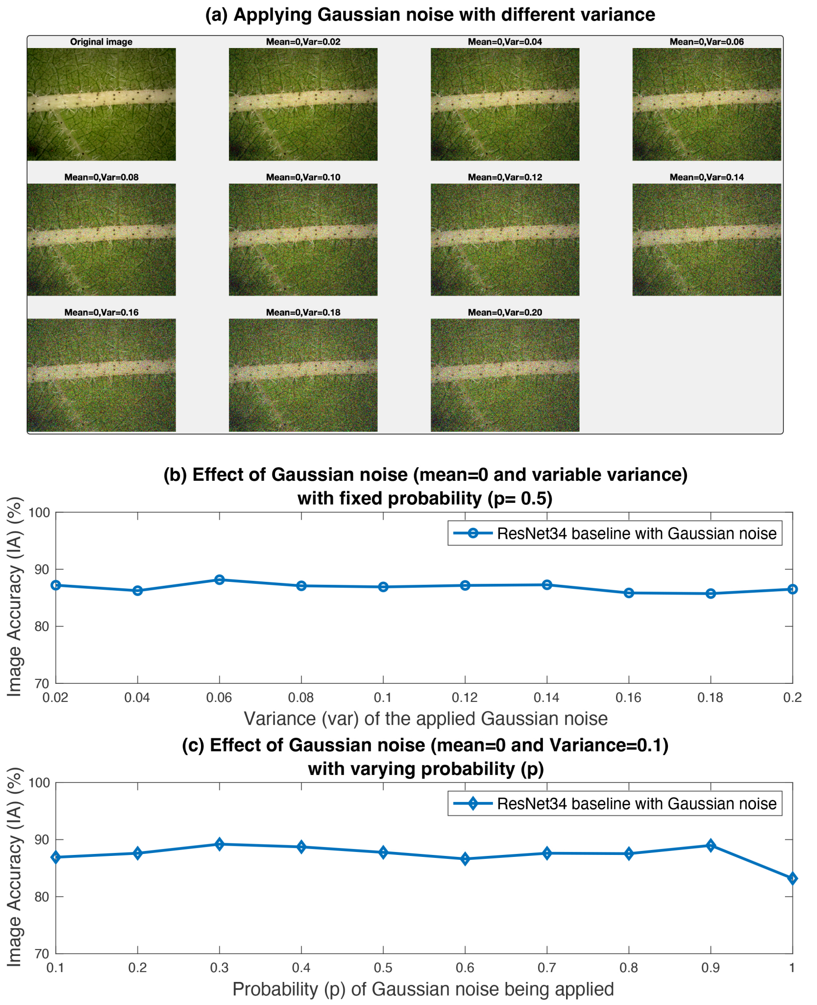

Additional file: Supplementary Figure 5
Effect of Gaussian noise on model accuracy.
Zero mean Gaussian noise with different variance (var) and probability (p) was applied to the dataset. The variance reflects the intensity of the noise (i.e. the higher the var, the higher the noise intensity) and the probability reflects how likely a given training image is to be corrupted with noise (i.e. $p = 0$ means never and $p = 1$ means always). Sub figure (a) shows the same image with increasing levels of Gaussian noise (i.e. increasing var). Sub figure (b) reports the model accuracy (Image Accuracy [IA]), with fixed $p = 0.5$ (i.e. 50% of training images were corrupted with noise), with Gaussian noise of different intensities. HairNet performed robustly even when subjected to severe Gaussian noise ($mean = 0, var = 0.2$). Sub figure (c) reports model accuracy (IA), with a fixed level of Gaussian noise ($mean = 0, var = 0.1$) and varying p. HairNet performed robustly up to $p = 0.9$ (i.e. 90% of the training images were corrupted), and dropped at $p = 1$ at which point the training set became out of distribution with the testing set. Except for this extreme scenario, HairNet was found to be robust against Gaussian noise.

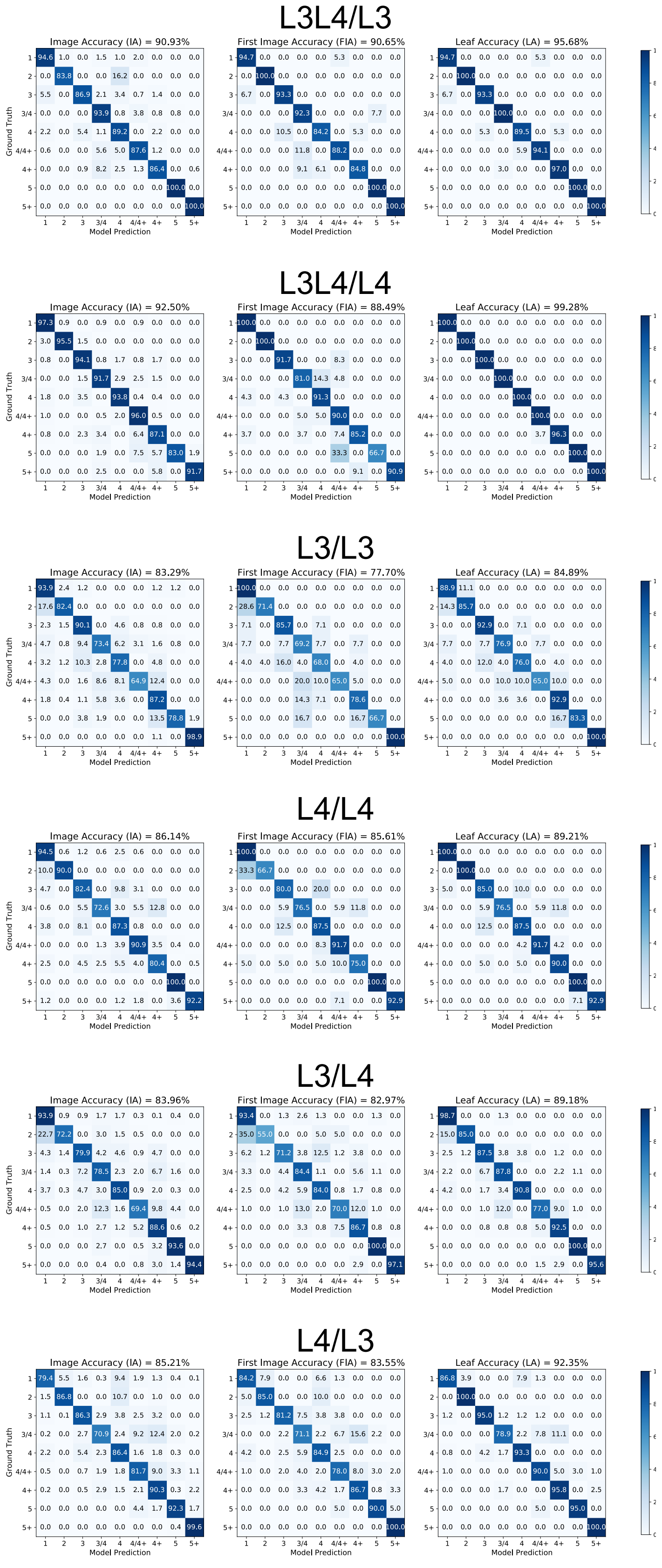

Additional file: Supplementary Figure 6
Normalized confusion matrix of HairNet predictions on leaf-based splits.

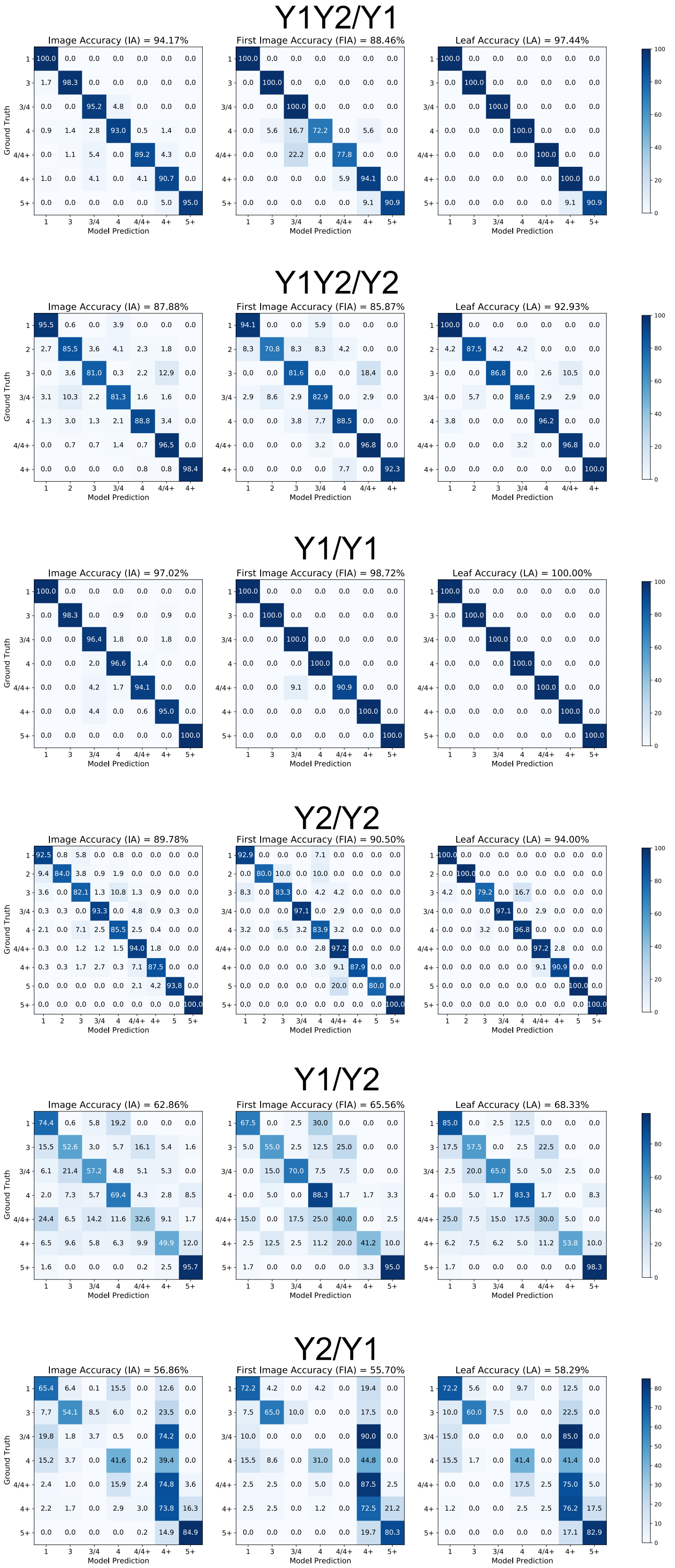

Additional file: Supplementary Figure 7
Normalized confusion matrix of HairNet predictions on year-based splits.

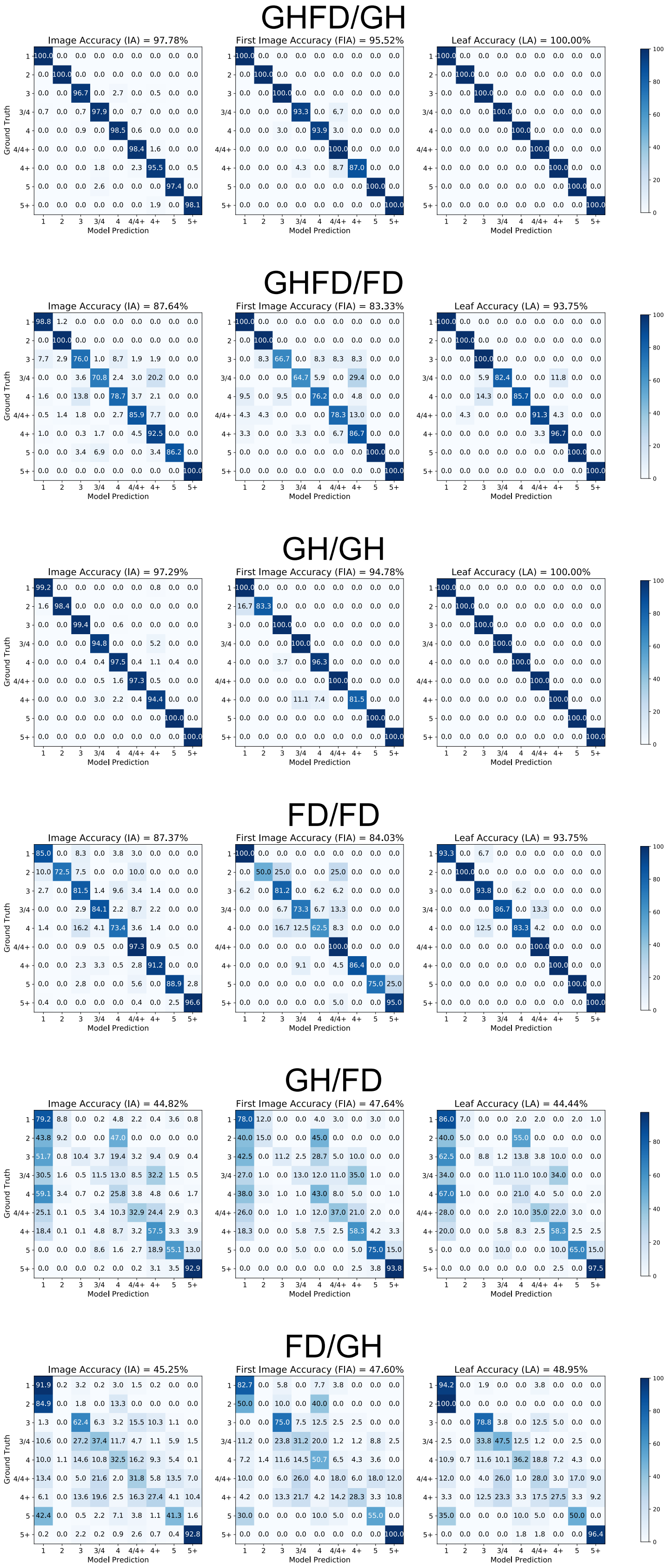

Additional file: Supplementary Figure 8
Normalized confusion matrix of HairNet predictions on environment-based splits.
